# Thrombogenic Catheter-Associated Superior Vena Cava Syndrome

**DOI:** 10.1155/2013/793054

**Published:** 2013-10-01

**Authors:** Imran Shaikh, Kenneth Berg, Nicholas Kman

**Affiliations:** Department of Emergency Medicine, The Ohio State University Medical Center, 750 Prior Hall, 376 W 10th Avenue, Columbus, OH 43210, USA

## Abstract

Superior vena cava syndrome has historically been associated with malignancy. With the increasing use of indwelling central lines, catheters, and pacemakers in the past decade, there have been an increasing number of cases associated with thrombosis rather than by direct external compression. Patients presenting to the ED with an acute process of SVC syndrome need to be assessed in a timely fashion. Computed tomography angiography (CTA) or magnetic resonance angiogram (MRA) are superb modalities for diagnosis and can quickly be used in the ED. Treatment is oriented towards the underlying cause of the syndrome. In cases of thrombogenic catheter-associated SVC syndrome, anticoagulation is the mainstay of treatment. We present a case report and discussion of a 56-year-old male with a history of metastatic colorectal cancer and an indwelling central venous port with acute signs and symptoms of superior vena cava syndrome.

## 1. Introduction

Superior vena cava (SVC) syndrome occurs when there is direct compression or obstruction of the superior vena cava. It is usually associated with a benign gradual increase of symptoms; however, in more acute scenarios, it can be life threatening. SVC syndrome is associated with malignancies like nonsmall-cell lung cancer, small-cell lung cancer, lymphoma, and metastatic lesions, which account for most cases [1]. Infections, such as tuberculosis, syphilis, histoplasmosis, and actinomycosis, have also been reported to cause this syndrome. Other causes include mediastinal fibrosis, vascular diseases, stenosis, and thrombosis. Clot-related SVC syndromes are generally more acute and are typically associated with central venous catheters and pacemaker leads. The increased use of indwelling lines and pacemakers in recent years has also yielded more cases of SVC syndrome overall. One report states that nonmalignant causes of SVC syndrome may represent up to 40% of cases [2]. It is important to consider causes other than direct compression by tumors, especially in more acute cases of SVC syndrome.

## 2. Case Presentation

**Figure 1:**
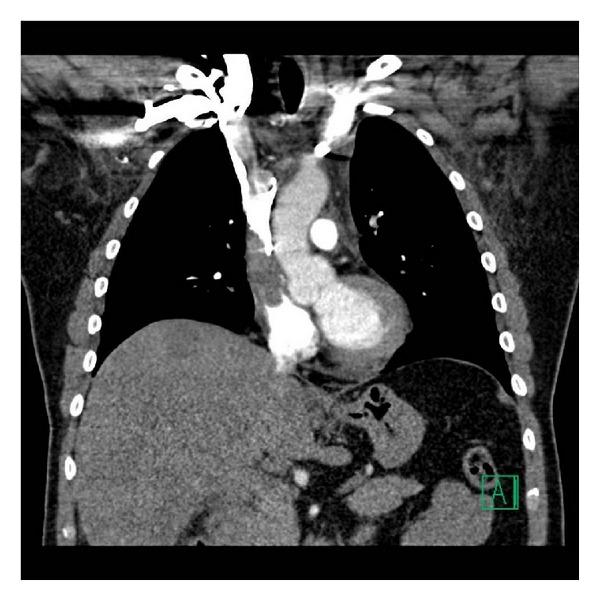
CT scan showing a thrombus extending from the internal jugular vein to the right atrium.

A 56-year-old Caucasian male presented to community emergency department with dyspnea, headache, chest congestion, and sore throat. The patient was recently diagnosed with metastatic colon cancer 2 months prior to his presentation in the ED, and he was currently undergoing chemotherapy. He had a right central venous access port placed one month before the onset of his symptoms. A CT scan of the chest was obtained and revealed a thrombus extending from the internal jugular to the right atrium (Figure 1). Patient was started on a heparin drip and transferred to our emergency department for further care. Upon arrival, the patient complained of shortness of breath, hoarseness, dysphagia without odynophagia, and neck swelling. His review of systems was negative for confusion, stridor, palpitations, lower extremity swelling, orthopnea, and cough. His past medical history includes colon cancer, hypertension, hyperlipidemia, and a cervical herniated disc that was surgically repaired many years prior to this ED visit. He smokes tobacco but does not use alcohol or any other illicit drugs.

**Figure 2:**
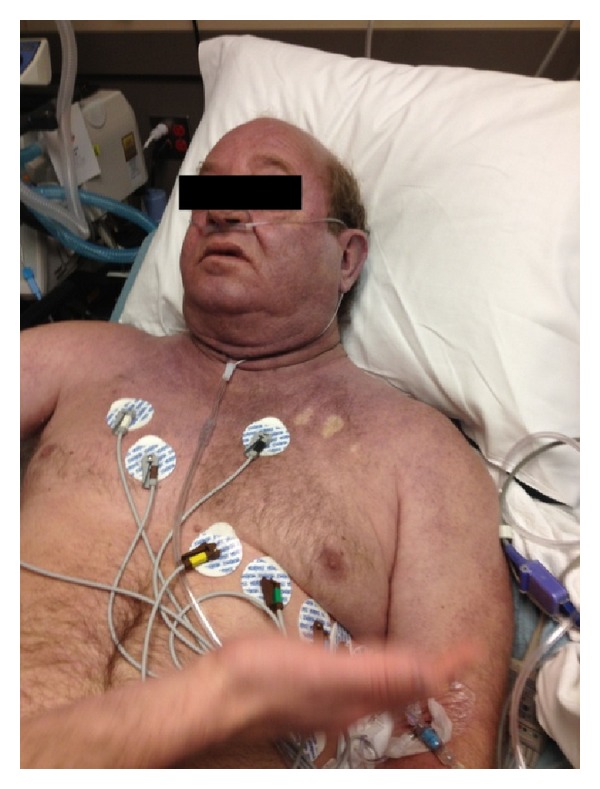
Patient's skin blanching after compression (author KB's hand in forefront). Note the purplish hue and neck edema.

 On presentation, the patient was tachypneic and in moderate respiratory distress. He was afebrile with a blood pressure of 118/85, heart rate of 98, respiratory rate of 24, and oxygen saturation of 96% on room air. On examination, he was fully alert and oriented. There were swelling in his neck and substantial plethora of his face, neck, and shoulders. His skin displayed a purplish hue that blanched after compression (Figure 2). He was visibly in a moderate respiratory distress, had muffling of his voice, and auscultation revealed bibasilar rales. There were no carotid bruits, jugular venous distention, stridor, drooling, trismus, or tracheal deviation. He had a normal complete blood count and basic metabolic panel. He was not on any blood thinners, and his INR initially was 1.4.

 By the time he arrived to our emergency department, he had already improved notably compared to his presentation to the community hospital, presumably, because of the heparin drip. Although he arrived on a nonrebreather mask, this was weaned off to nasal cannula fairly quickly as the patient continued to improve. An MRI was scheduled to evaluate for any brain metastases secondary to the need for focused thrombolysis. Vascular surgery was consulted and decided to attempt directed thrombolytic therapy. An interventional radiologist performed an SVC venogram and placed a tPA infusion catheter through the brachial veins bilaterally. A repeated bilateral upper extremity central venogram showed a residual but nonocclusive thrombus in the bilateral innominate veins approximately 12 hours later. Venograms with bilateral venoplasty were performed a day later and showed stenotic bilateral innominate veins. An infusion of tPA ran continuously for 48 hours. The procedure was uncomplicated, and the patient tolerated it well. At the time of discharge, the patient's symptoms were all remarkably improved. He was sent home on warfarin after an anticoagulation bridge with enoxaparin.

## 3. Discussion

 The superior vena cava is the primary vessel for blood return from the head, neck, and upper body. There are many reasons why the SVC might be obstructed. First, the vessel is thin-walled with low venous pressures which portends to a thrombogenic diathesis. Also, the vein is anatomically constrained, making it easily compressible by nearby structures. Lymph nodes surrounding the SVC can become enlarged and can externally compress the vein in mediastinal diseases [3]. There are several risk factors associated with catheter-related thromboses. Placement of a central catheter can damage the endothelial wall. This risk increases even more when the catheter is incorrectly placed. Catheters can also impede blood flow within a vein, causing blood stasis. Along with hypercoagulability, these are the components of Virchow's triad, which are the three factors considered to contribute to thrombosis [4].

 When the SVC becomes obstructed, collateral venous return to the heart develops primarily via the azygos venous system. With slowly progressive SVC obstructions, such as those caused by tumor compression, adequate collateral venous return develops and minimizes the sequelae of the syndrome. In a more acute obstruction, as seen with thrombosis, the presentation can be much more life threatening [2]. The incidence of SVC syndrome due to colorectal cancer is extremely rare, with reports linking these two only with the rare chance of metastasis to the mediastinal lymph nodes [5]. Given the acute presentation of our patient's symptoms, the low incidence of SVC syndrome due to colorectal cancer, and the catheter-related risk factors, the most likely etiology of this case is from the catheter. 

 Common signs and symptoms of SVC syndrome include swelling of the face, neck, and upper extremity, associated with dyspnea, cough, and plethora. The classic appearance of facial edema, flushing, and jugular venous distention which is exacerbated by having the patient's arms raised over the head is known by the eponym “Pemberton's sign” [6]. Other findings can include dysphagia, odynophagia, hoarseness, stridor, fatigue, periorbital edema, chest pain, headache, changes in mental status, and syncope. Diseases such as congestive heart failure, angioedema, and constrictive pericarditis may present with similar clinical manifestations and should remain in the differential diagnosis when evaluating these patients [7].

 SVC syndrome can be diagnosed clinically with a detailed history and physical exam. However, imaging is needed for a definitive diagnosis. Ultrasound may be used initially, and it is quick, inexpensive, and does not involve radiation or contrast dye. It has a high sensitivity towards peripheral upper extremity thromboses. However, it has limited capabilities in visualizing the central subclavian and brachiocephalic veins. This is in part due to the clavicle causing an acoustic shadow. Contrast venography can confirm the diagnosis of SVC syndrome and is also necessary in order to direct treatment and monitor progression of therapy. Contrast venography has several drawbacks, however. It uses iodine agents, which can cause nephrotoxicity or an allergic reaction. In the setting of obstruction, difficulty in cannulation occurs during this procedure. In our case, venous cannulation for contrast venography was considerably challenging. Therefore, in the ED, magnetic resonance angiography (MRA) and computed tomography angiography (CTA) were the preferred imaging modalities, as they provided an accurate diagnosis, were noninvasive, and generally have minimal complications. They give similar detail as venography and can help with evaluating the extent of obstruction. Formation of collateral vessels seen either in CTA or MRA can indicate SVC syndrome with a sensitivity and specificity of 96% and 92%, respectively [4].

 In any patient that presents with SVC syndrome, the goal is to treat the underlying cause. The type of treatment depends on the etiology and severity of symptoms. If there are signs or suspicion of airway compromise, intubation should be performed without delay. When a thrombus is the reason for obstruction, anticoagulation is the mainstay of therapy with thrombolytics used as adjuncts [7]. As there have not been any randomized controlled studies evaluating the treatment of upper extremity thromboses, dosing should follow treatments of pulmonary embolism or lower extremity deep vein thrombosis. Initial therapy should be started with parenteral anticoagulants such as heparin and followed with warfarin or low molecular weight heparin (LMWH). In upper extremity thromboses, there have been no studies comparing the differences between warfarin and LMWH [8, 9]. Balloon angioplasty with stenting can also be used as first line therapy to provide prompt symptomatic relief in cases, where SVC syndrome is caused by a thrombus or stenosis via catheter. It can also be considered for refractory SVC syndrome, although the long-term outcomes from doing this have not been extensively studied. When SVC syndrome is caused by malignancy, radiation and chemotherapy should be considered, as tumor burden would decrease and potentially resolve the symptoms. Radiotherapy should only be done after a biopsy is performed, and chemotherapy is preferred in chemosensitive tumors. Clinical trials have shown that the traditional methods of elevating the head of the bed, dexamethasone, and furosemide have no benefit [7].

 Superior vena cava syndrome has historically been associated with malignancy. With the increased use of indwelling central lines and pacemakers in the past decade, there have an increasing amount of thrombogenic and stenotic SVC syndrome cases. These patients usually present with more acute sequelae because of the relatively rapid rate of thrombogenesis and lack of venous collateral development compared to a slowly progressing obstruction associated with malignancy. Patients presenting to the ED with an acute process of SVC syndrome need to be assessed in a timely fashion. CTA or MRA are superb modalities for diagnosis and can quickly be used in the ED. Treatment is oriented towards the underlying cause of the syndrome. In our case, thrombogenic catheter-associated SVC syndrome, anticoagulation is the mainstay of treatment. Thrombolytics and balloon angioplasty with stenting have been shown to be beneficial when prompt management of symptoms is needed.
